# Cattle brucellosis in traditional livestock husbandry practice in Southern and Eastern Ethiopia, and its zoonotic implication

**DOI:** 10.1186/1751-0147-53-24

**Published:** 2011-04-07

**Authors:** Bekele Megersa, Demelash Biffa, Fekadu Niguse, Tesfaye Rufael, Kassahun Asmare, Eystein Skjerve

**Affiliations:** 1 School of Veterinary Medicine, Hawassa University, P.O. Box 05, Hawassa, Ethiopia; 2 Center for Epidemiology and Biostatistics, Norwegian School of Veterinary Science, P.O. Box 8146 Dep., 0033 Oslo, Norway; 3 National Animal Health, Diagnostic and Investigation Centre, P.O. Box 04, Sebeta, Ethiopia

## Abstract

**Background:**

Cattle brucellosis has significant economic and zoonotic implication for the rural communities in Ethiopia in consequence of their traditional life styles, feeding habits and disease patterns. Hence, knowledge of brucellosis occurrence in traditional livestock husbandry practice has considerable importance in reducing the economic and public health impacts of the disease.

**Methods:**

A total of 1623 cattle sera were serially tested using the rose Bengal test as screening and complement fixation test as confirmatory tests. The Stata survey command was used to establish prevalences for the overall and individual variables, while potential risk factors for seropositivity were analyzed using a multivariable logistic regression analysis.

**Results:**

The results showed that 3.5% (95% CI = 2.4, 4.5%) of the animals and 26.1% (95% CI = 18.6, 33.7) of the herds tested had antibodies against Brucella species. Village level seroprevalence ranged from 0% to 100%. A higher seroprevalence was observed in pastoral system than mixed farming although this variable was not significant in the final model. The final logistic regression model identified herd size; with large (odd ratio (OR) = 8.0, 95% CI = 1.9, 33.6) and medium herds (OR = 8.1, 95% CI = 1.9, 34.2) showing higher risk of Brucella infection when compared to small herds. Similarly, the odds of Brucella infection was higher in cattle aged above 4 years when compared to age groups of 1-2 (OR = 5.4, 2.1, 12.9) and 3-4 years (OR = 3.1, 95% CI = 1.0, 9.6). Herd level analysis of the risk factors revealed that large and medium herds as well as herds kept with multiple livestock species were at higher risk of acquiring Brucella infection. Brucellosis in traditional livestock husbandry practices certainly poses a zoonotic risk to the public, in consequence of raw milk consumption, close contact with animals and provision of assistance during parturition. Due to lack of diagnostic facilities and information on its occurrence, human brucellosis is most likely misdiagnosed for other febrile diseases prevailing in the areas and treated empirically.

**Conclusions:**

The results of this study demonstrated that bovine brucellosis is widely prevalent in the study areas particularly in pastoral production system. Hence, the study suggests the need for implementing control measures and raising public awareness on prevention methods of brucellosis.

## Introduction

Brucellosis remains widespread in the livestock populations, and represents a great economic and public health problem in African countries. Brucellosis causes abortion which is the major means of spread by infected afterbirth or fetus as well as excretion of excessive organisms which can easily be acquired by susceptible animals. The epidemiology of the disease in livestock and humans as well as appropriate preventive measures are not well understood, and in particular such information is inadequate in sub-Saharan Africa [1]. The epidemiology of cattle brucellosis is complex and influenced by several factors [2]. These can be broadly classified into factors associated with the transmission of the disease between herds, and factors influencing the maintenance and spread of infection within herds. The climatic and agro-ecological diversities of Ethiopia may allow a wide range of livestock production systems, and therefore, different management systems, multiple livestock species per holding, stock density and social organizations to handle livestock may account for the widespread risk factors for maintenance and transmission of cattle brucellosis.

The evidences of *Brucella *infections in Ethiopian cattle have been serologically demonstrated by different authors [3-7]. A relatively high seroprevalence of brucellosis (above 10%) has been reported from smallholder dairy farms in central Ethiopia [4] while most of the studies suggested a low seroprevalence (below 5%) in cattle under crop-livestock mixed farming [3,6,8,9]. There is a scarcity of published literature on the status of cattle brucellosis in pastoral areas of the country where large population of cattle are reared. So far, a study carried out in east Showa zone of Ethiopia showed a relatively higher seroprevalence in pastoral than agropastoral system [10].

Most of the previous studies on cattle brucellosis have been carried out in central and northern Ethiopia, and do not provide an adequate epidemiological picture of the disease in different agro-ecological zones and livestock production systems of the country. In particular, there is no information on cattle brucellosis across various livestock production systems of southern and eastern part of the country, which gave impetus to the initiation of this study. The present study was therefore aimed at determining the prevalence of cattle brucellosis and associated risk factors across the two livestock production systems, pastoral and crop-livestock mixed systems, in Southern and Eastern Ethiopia.

## Materials and methods

### Study area and study animals

**Figure 1:**
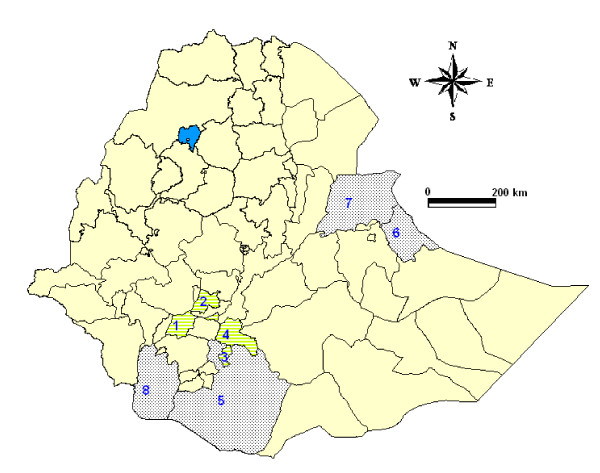
**Zonal administrative map of Ethiopia showing the study areas: Zones indicated by numbers 1-4 (1. Dawro, 2. Hdiya, 3. Gedeo, 4. Sidama) are mixed farming while the remaining zones are pastoral (5. Borana, 6. Jijiga, 7. Shinle and 8. South Omo)**.

The study was carried out in eight administrative zones in southern and eastern Ethiopia; namely Dawro, Sidama, Gedeo, Hadiya, South Omo, Borana, Jijiga and Shinle (Figure 1). A total of 33 districts were selected from these zones, from which 96 villages were chosen for sampling. The study areas are generally characterized by diverse agro-climatic zones with altitude ranging from 370 meters in Dasanach and Omoratte districts (South Omo) to 3175 meters above sea level (m.a.s.l.) in Bulle district (Gedeo). Based on altitude range, the study areas were broadly classified into the traditional agro-climatic classifications of lowland "*Kola" *(< 1500 m.a.s.l.); midland "*Weynadega" *(1500 - 2400 m.a.s.l.) and highland "*Dega" *(> 2400 m.a.s.l.). Geographically, the study areas cover latitude and longitude ranges of 03° 34'21" to 10° 54' 93" East and 36° 01' 50" to 43° 70' 56" North.

Livestock production in the area is dominated by extensive production system, in which indigenous cattle are allowed to graze freely during day time and kept in open enclosures during the night. The extensive production system is further categorized into pastoral and crop-livestock mixed farming systems. Four zones; Borana, Jijiga, Shinle and South Omo are characterized by pastoral system while the remaining four zones practice crop-livestock mixed farming. The number and composition of animal species per holding in the mixed farming is relatively lower than the pastoral system. Table 1 shows the mean herd size and sample proportion over administrative zones. Livestock composition varies from keeping cattle as dominant stock with variable number of small ruminants in crop-livestock mixed farming systems to a camel, cattle and small ruminant composition in pastoral areas. As livestock brucellosis control intervention by immunization has never been attempted in Ethiopia, there is no history of vaccination against brucellosis in the study areas.

**Table 1:** Mean herd size and sample proportions of the studied herds in each administrative zone of the study areas

Production systems	Administrative Zones	Herd size Mean (95% CI)	Sample proportion (%)
Mixed farming*	Dawro	8.8 (8.4, 9.3)	95.4
	Gedeo	12.6 (11.8, 13.4)	83.3
	Hadiya	15.4 (14.9, 15.9)	87.0
	Sidama	13.1 (12.7, 13.5)	91.0
Pastoral system	Borana	43.6 (42.5, 44.7)	49.4
	Jijiga	20.1 (19.4, 20.9)	77.2
	Shinle	20.7 (19.9, 21.4)	70.0
	South Omo	45.2 (44.0, 46.3)	43.2

* Mixed farming, also known as sedentary farming, is where crop-livestock mixed farming is practiced

### Study Design and sample size determination

A cross-sectional multi-stage sampling, with zone as highest and herd as lowest sampling stages, district and village in between the two stages, was carried out from October 2007 to March 2008. Selection of the study unit at each stage was based on a mixed design of convenience and random samplings. Zones were conveniently selected based on geographic localities and dominant livestock production system, whereas districts and villages were randomly selected following a randomization of districts and villages when lists were obtained from respective administratives. When this was not the case in pastoral systems, herds were sampled conveniently in consultation with herd owners. As information on prevalence of cattle brucellosis is not available for the study areas, we adopted a sampling technique for detection of disease [11]. Assuming a target prevalence of 5% and district level sensitivity at 95%, we would need to sample a minimum of 59 animals to get at least one positive animal. With available logistics and resources, we managed to sample a total of 1623 animals from 33 districts. The number of animals sampled from each area could vary according to livestock density, access to transportation and availability of logistic facilities. Study animals include all animals aged 1 year and above in a selected herd (while about 50% of the animals in large herds were to be sampled).

### Serum Sample Collection and Testing

From each animal, 10 ml of blood was aseptically collected from the jugular vein using plain vacutainer tubes and clotted at room temperature for 12 hours. Sera were then collected in sterile tubes and transported to the laboratory using ice box where stored at -20°C until tested. Subsequently, the rose Bengal test (RBT), Institut Pourquir, rue de la Galera 34097 Montpellier, France, was carried out by adding an equal volume of antigen (30 μl) and serum onto the glass slide. The antigen and test serum were mixed thoroughly by plastic applicator, shaken for 4 minutes, and degree of agglutination was visually recorded immediately. Complement fixation test (CFT) was performed at the National Animal Health Diagnostic and Investigation Center (NAHDIC), Sebeta Ethiopia, using *Brucella *antigen and control sera (positive and negative) produced by Veterinary Laboratories Agency (VLA, New Haw Addlestone, Surrey, KT15 3NB, UK). The antigen was standardized at 1:10 dilution. Two-fold dilutions of test sera (1:5, 1:10, 1:20 and 1:40) were prepared in U-shape 96-well micro-titer plates before adding *Brucella *antigen, guinea pigs complement and 3% sensitized sheep red blood cells. The plates were incubated at 37°C for 30 minutes with agitations (warm fixation) and results were read after the plates have been centrifuged at 2500 rpm for 5 minutes at 4°C. CFT was regarded positive when the reading was as complete fixation (complete inhibition of haemolysis) or nearly complete fixation (25% haemolysis) at 1:10 dilutions. This cut-off point was taken to optimise specificity and ensure that seropositive cases were due to brucellosis. This cut-off is routinely used by NAHDIC in their diagnostic system. An animal was considered positive if tested seropositive on both RBT and CFT in serial interpretation. The test was regarded as valid if the negative control serum showed complete haemolysis and the positive control shows inhibition of haemolysis. The use of RBT/CFT combinations, the most widely used serial scheme, is generally recommended to maximize specificity of the test result by ruling out false positive serological cross-reactions [11].

### Data collection and analysis

Putative biological and environmental factors believed to be associated with the epidemiology of brucellosis were recorded in a Microsoft Excel^® ^Spread Sheet. Data on individual animals such as sex, age, herd size, stock composition, production system and agro-climate were recorded. All the necessary statistical analysis was performed using STATA version 10.0 for Windows (Stata Corp. College Station, TX). The individual positive outcome was defined as any animal with RBT+ and CFT+, while herd or village positivity was any herd or village having at least one seropositive animal.

The prevalence of *Brucella *antibodies at the individual level was established by the Stata *survey *command considering village as a primary sampling unit and each variable as a stratum and sampling weight variable. Association of exposure variables with seroprevalence was analyzed at individual animal level using logistic regression following adjustment for sampling weight according to sampled numbers and estimated number of animals in each village. A multivariable logistic regression model was used to identify risk factors associated with *Brucella *infection, at individual and herd levels, keeping village as the cluster variable. Variables with a p-value lower than or equal to 0.25 (in univariable analysis) were included in the multivariable logistic model. Further selection of variables was based on backward elimination procedure using a LR-test at 0.05 as cut-point. Prior to building a final model, variables were tested for interaction effects using cross-product terms and for multiple-collinearity using the collinearity matrix index. The validity of the model to the observed data was assessed by computing the Hosmer-Lemeshow goodness-of-fit test. Finally, deviant covariate patterns and their influences on parameter estimates of the model were identified.

## Results

The test results show that 63 of the tested animals were positive for RBT, of which 51 (81.0%) were further confirmed to be seropositive by CFT. The overall seroprevalence records were 3.5% (95% CI: 2.4, 4.5%), 26.1% (95%CI: 18.6, 33.7), and 31.3% (95%CI: 22.4, 41.6) at animal, herd and village levels, respectively. The seroprevalence distribution of *Brucella *infection at animal, herd and village levels in the study areas is presented by Table 2. The results of herd and village seroprevalence are nearly comparable. This could result from clustering effects at village levels and thus village would be more appropriate unit of the study than herd. Village level seroprevalence ranged from 0% to 100% with higher seroprevalences in pastoral systems. The highest village level seroprevalence (100%) was recorded for Borana, whereas seroprevalences of over 40% were recorded for villages in Jijiga and Shinle pastoral areas of Eastern Ethiopia. The seroprevalence was generally low in mixed farming areas of Sidama and Gedeo zones, while no seropositive case was detected in villages of Dawro zone.

**Table 2:** Distribution of seropositivity (%) to *Brucella *antigens in indigenous cattle (at different levels) across the study areas

Study areas		Animal level*		Herd level		Village level	
**Production system**	**Zones**	**No of animals**	**Prevalence** **(95% CI)**	**No of herds**	**Prevalence** **(95% CI)**	**No of Villages**	**Prevalence** **(95% CI)**

Mixed farming	Dawro	104	0 (-)	13	0 (0)	7	0 (0)
	Gedeo	161	0.5 (0.05, 1.5)	17	5.9 (0.3, 30.8)	10	10 (0.5, 45.9)
	Hadiya	245	3.5 (1.1, 5.8)	20	30.0 (12.8, 54.3)	17	35.3 (15.3, 61.4)
	Sidama	390	1.8 (0.4, 3.0)	37	13.5 (5.1, 29.6)	26	19.2 (7.3, 40.0)
Pastoral system	Borana	271	4.7 (2.1, 7.3)	16	68.8 (41.5, 87.9)	6	100.0
	Jijiga	62	3.0 (1.1, 7.1)	4	50.0 (9.2, 90.8)	4	50.0 (9.2, 90.8)
	Shinle	210	6.6 (3.1, 10.1)	15	40.0 (17.1, 67.1)	14	42.9 (18.8, 70.4)
	South Omo	180	3.4 (0.9, 6.1)	12	33.3 (11.3, 64.6)	12	33.3 (11.3, 64.6)

Total		1623	3.5 (2.4, 4.5)	134	26.1 (19.1, 34.5)	96	31.3 (22.4, 41.6)

* Animal level seroprevalence was calculated following adjustment for sample weight

Table 3 presents results of animal level univariable analysis showing the association of the exposure variables and *Brucella *seropositivity. The results showed that most of the recorded variables showed a high degree of association with seropositivity to *Brucella *infection.

**Table 3:** Prevalences (%) and univariable analysis of the potential risk factors for seropositivity to *Brucella *antibodies in indigenous cattle (following adjustment for sampling weight)

Variable	Level	No. of Sample	Prevalence (95% CI)	OR (95% CI)	P-value*
Age groups	1 - 2 years	497	1.0 (0.1, 1.8)	1.0 (-)	
	3-4 years	366	3.0 (1.0, 5.0)	3.1 (1.0, 9.3)	0.046
	> 4 years	760	5.1 (3.4, 7.0)	5.4 (2.1, 14.1)	0.001
Sex	Male	485	2.2 (0.7, 3.3)	1.0 (-)	
	Female	1138	4.1 (2.8, 5.4)	2.1 (1.0, 4.3)	0.040
Herd size	< 15 animals	414	0.5 (0.2, 1.2)	1.0 (-)	
	15 - 29 animals	758	3.9 (2.1, 5.6)	8.1 (1.9, 34.7)	0.005
	≥ 30 animals	451	4.3 (2.5, 6.1)	9.1 (2.2, 38.3)	0.003
Agro-climate	Low altitude	435	4.4 (1.8, 7.0)	1.0 (-)	
	Mid altitude	1008	3.3 (2.2, 4.4)	0.7 (0.4, 1.5)	0.391
	High altitude	180	1.0 (0.3, 2.4)	0.2 (0.1, 9.5)	0.043
Production system	Mixed farming	900	1.8 (0.8, 2.8)	1.0 (-)	
	Pastoral system	723	4.5 (2.9, 6.1)	2.6 (1.3, 5.1)	0.007
Species composition	Cattle-small rum	1080	2.4 (1.1, 3.6)	1.0 (-)	
	Cattle-small rum-camel	271	4.7 (2.9, 6.5)	2.0 (1.0, 4.0)	0.041
	Camel-cattle-small rum	272	5.8 (2.2, 9.5)	2.6 (1.1, 6.1)	0.032

* Variables with p ≤ 0.25 identified as possible risk factors and offered to multivariable model, rum: ruminant

Variables with a p-value <0.25 from univariable analysis were included in the final multivariable logistic model. Two variables, sex and altitude range that showed collinearity with other variables (sex with age, altitude with production system, livestock composition and herd size), were not included in the multivariable logistic regression model. The rest variables; age, herd size, stock composition and production system were offered to the model. Further selection of variables in the final model was based on stepwise backward elimination procedure.

The final multivariable logistic regression model (Table 4) showed that animals kept in large (OR = 8.1, 95% CI = 1.9, 34.2) and medium (OR = 8.0, 95% CI = 1.8, 35.0) herd sizes were more likely to be exposed to *Brucella *infections than those maintained in small herds. Similarly, animals above 4 years of age were more likely to acquire infections than those in age groups of 1-2 (OR = 5.4, 2.1, 12.9) and 3-4 years (OR = 3.1, 95% CI = 1.0, 9.6). Herd level analysis of the risk factors identified an increase in herd size and ruminant composition as the major risk factors for herds to acquire *Brucella *infection. The Hosmer-Lemeshow goodness-of-fit test showed that the model fitted the data well (*χ*^2 ^= 2.7, P = 0.61). Post-estimation statistics didn't identify any covariate patterns (observations) that showed an outlying distribution and any influence on parameter estimates of the model.

**Table 4:** Multivariable logistic regression model of risk factors for *Brucella *seropositivity in cattle at individual (n = 1623) and herd (n = 134) levels using village as the cluster variable

Variable	level	Odds Ratio	95% CI	P-value
Individual animal level				

Age	1 - 2 years	1.0	-	-
	3-4 years	3.1	1.0, 9.6	0.045
	> 4 years	5.2	2.1, 12.9	0.000
Herd Size	< 15 animals	1.0	-	-
	15 - 29 animals	8.1	1.9, 34.2	0.005
	≥ 30 animals	8.0	1.8, 35.0	0.006

Herd level				

Species composition	Cattle-small rum	1.0	-	-
	Cattle-small rum-camel	2.7	1.1, 6.8	0.039
Herd Size	< 15 animals	1.0	-	-
	15 - 29 animals	11.3	2.4, 51.9	0.002
	≥ 30 animals	19.6	3.8, 100.9	0.000

## Discussion

The study showed that antibodies to *Brucella *infection were prevalent across the study areas except for Dawro where all tested animals (n = 104) were seronegative (Table 2). The overall animal level seroprevalence of 3.5% was comparable with the findings of other authors in Ethiopia; 3.2% by Berhe et al. [3], 4.6% by Hailemelekot et al. [8], 3.1% by Ibrahim et al. [6], 2.9% by Jergefa et al. [5] and 4.9% by Mekonnen et al. [7]. Similarly, comparable seroprevalences were reported from some other countries: 4.2% in Eritrea [12], 3.3% in Central Africa [13] and 5.8% in Nigeria [14]. Our finding of 26.1% herd seroprevalence is similar to 24.1% reported by Mekonnen et al. [7] whereas most of the other studies in Ethiopia showed a relatively low seroprevalence [5,6,9]. Conversely, higher herd level seroprevalences have been recorded by other authors; 62% from Zambia [15], 55.6% from Uganda [16] and 42.3% from Ethiopia [3]. Such contrasting findings could be either related to the overall animal level prevalence status of the disease or number of animals per the studied herds (herd size). The effect of an increased number of animals per herd was also observed in a specific finding of this study (68.8%: higher herd level seroprevalence in Borana than others without much difference in individual animals). This large herd effect reflects the larger numbers of samples in larger herds.

Higher herd and village levels seroprevalences were observed in pastoral production systems, when compared to crop-livestock mixed framings, similar to what has already been demonstrated by earlier researchers [1,10,12,16]. This is mainly attributed to the nature of pastoral production system: high herd mobility, multiple livestock species herding and increased number of animals per holdings. The settlement pattern of pastoral community in Ethiopia is characterized by clustering of households with close proximity of herds in the pastoral camps. Additionally, pastoral households often keep a diverse composite of livestock species as part of a coping mechanism for uncertainties and risks. Such conditions certainly increase aggregation and interaction of different animals at villages, grazing fields and water points, thus, facilitate transmission of the disease. The dynamics and frequent migration of pastoral herds might increase the chance of coming into contact with other potentially infected herds and exposure to geographically limited or seasonally abundant diseases. Mobility also increases the opportunity of interactions with wild animals. This has already been confirmed by Muma et al. [15] in that herds coming into contact with wildlife had higher likelihood of acquiring infection than those without contact.

The lowest seroprevalence was recorded in mixed farming areas of Sidama and Gedeo zones while no positive case was detected in Dawro zone. These areas are partly cash crop (coffee, fruits, vegetables, spice) growing region of the country where small numbers of animals are kept separately. In some cases, animals are tethered around farmland or homestead and feed on post harvest products of the farms, a condition which decreases mobility and contact between herds. Similar findings of low seroprevalences were reported from crop-livestock mixed farming areas of Eritrea [12] and Ethiopia [3]. Likewise, absence of seropositive animal in Dawro may be due to a small sample size coupled with low prevalence of brucellosis in mixed farming area.

The multivariable logistic analysis identified herd size, age group (animal level) and livestock species composition (herd level) as risk factors for acquiring *Brucella *infection (Table 4). The higher seropositivity observed in the large herds is in accordance with previous findings [3,7,15] and can be explained by the fact that an increase in herd size is usually accompanied by an increase in stocking density, one of the determinants for exposure to *Brucella *infection especially following abortion or calving [2]. Risks linked to herd size and livestock species composition was observed in final model of herd level analysis. Herds kept with multiple livestock species had higher odds of seropositivity to Brucella infection, suggesting possibilities of cross-species transmission of *Brucella *infection. Multiple livestock species herding, keeping of small ruminants along with cattle or camels, has been reported as risk factor for seropositivity to *Brucella *infections [17,18].

Association of age with seropositivity to *Brucella *infection is consistent with the findings of earlier studies [3,4,6-8]. Age is one of the intrinsic factors which influences the susceptibility to *Brucella *infection. Brucellosis appears to be more associated with sexual maturity [19], and higher seroprevalence is repeatedly reported in sexually mature animals. Seroprevalence may increase with age as a result of prolonged duration of antibody responses in infected animals and prolonged exposure to pathogen, particularly in traditional husbandry practice where females are maintained in herds over long period of time. In our data analysis, the fact that females showed higher seropositivity than male animals, and this variable (sex) showed collinearity with age may also substantiate this fact. In the study areas, female animals are maintained in herds over extended time period thus, have ample time for exposure to the pathogen and being source of infection for other animals. Hence, practice of culling breeding females with reduced reproductive performances and old age could reduce the risk of within herd spread of brucellosis and its zoonotic hazard to human.

Although developed countries have successfully controlled brucellosis, many developing countries such as Ethiopia, have not been able to react adequately and the disease continues to be a major public and animal health problem. Control and eradication of brucellosis is almost exclusively based on the serological testing of animals and the subsequent culling of those that are seropositive for antibodies to *Brucella *species [20,21]. As no single serological test is appropriate in all epidemiological situations, the use of two tests applied serially is usually recommended for maximal specificity and ruling out false positive cross-reactions [20,21]. A combination of RBT and CFT tests is the most widely used serial testing scheme. Selection of RBT as screening test is based on cost, easy performance and high sensitivity, especially in endemic areas [15]. The second test, CFT is selected due to its high specificity to discriminate between false positive cross-reactions and *Brucella *infections [20]. When test specificities are conditionally independent of each other, the resulting expected specificity of serial testing is said be higher than the corresponding individual specificities of each test [11]. Conversely, serial testing using pairs of specificity-correlated serological tests (RBT, CFT, c-ELISA) has been argued, in favor of a highly specific single test such as i-ELISA, to have lower specificity than expected when applied to disease free population [22]. When such test is applied to a low disease prevalence (below 1%) or disease free population, the predictive value of the test drops closer to zero and increased proportion of non-infected animals are classified as seropositive [21,22]. Therefore, consideration should be given to all factors that have impact on the relevance of the test method and test results to a specific diagnostic interpretation or an epidemiologic situation.

Adherence to traditional farming practices, preference for fresh dairy products and contact with animals have been reported to be risk factors for human exposure [23-26]. In our study area, close intimacy with livestock, low awareness on zoonotic importance of brucellosis, tradition to consume raw milk and pattern of the disease in animals may certainly increase the risk of human exposure to *Brucella *infections. Despite the widespread distribution of brucellosis in animals and ample exposure factors for humans in Ethiopia, only scanty published information is available regarding human brucellosis. According to these studies, there are large number of undiagnosed cases of febrile diseases, neurological complications, joint problems and certain generalized complications in rural communities that might be associated with brucellosis [9,24-26]. Seroprevalences of 34.9% and 29.4% have been reported from patients with fever of unknown origin in Borana and South Omo (Hamar) pastoral communities, respectively [24]. Similarly, a seroprevalence of 5.3% has been reported from limited number of animal health professionals, occupationally risk group, in Sidama zone of Southern Ethiopia [9]. These suggest that large number of undiagnosed cases with fever, neurological complications and other generalized complications in rural and pastoral communities are misdiagnosed and treated empirically as malaria or fever of unknown origin.

In conclusion, our study revealed that bovine brucellosis is widely prevalent in cattle herds of most villages of the study areas with higher seroprevalence in pastoral than mixed farming areas. Animals aged above 4 years, large herd size and herds kept mixed with more livestock species are at increased risk of acquiring *Brucella *infection. Hence, the need for implementing control measures and raising public awareness on zoonotic transmission of brucellosis are recommended.

## Competing interests

The authors declare that they have no competing interests.

## Authors' contributions

BM participated in the design, sampling, data analysis and write-up. FN and TR carried out sample collection, testing and writing. DB participated in data analysis and editions, while KA took part in the writing. ES involved in the design, data analysis and coordination. All authors read and approved the final manuscript.
